# *S100A14* Facilitates Pancreatic Cancer Progression via *S100A16*-Mediated *p53* Suppression

**DOI:** 10.32604/or.2025.070207

**Published:** 2026-02-24

**Authors:** Pingping Hu, Zhenhao Fei, Jianhua Bai, Zhiwen Wang, Yun Jin

**Affiliations:** 1 Department of Hepatopancreatobiliary Surgery, The First People’s Hospital of Yunnan Province, Kunming, 650032, China; 2 Department of Hepatopancreatobiliary Surgery, The Affiliated Hospital of Kunming University of Science and Technology, Kunming, 650032, China

**Keywords:** Pancreatic cancer, *S100A14*, *S100A16*, *p53*, tumor progression

## Abstract

**Objectives:**

Pancreatic cancer (PC) is characterized by poor prognosis due to its limited treatment choices and delayed detection. *S100A14* has been implicated in tumor progression, yet its regulatory hierarchy and functional interplay in PC remain unclear. This study aimed to define the role of *S100A14* in PC progression.

**Methods:**

Integrated bioinformatic analyses of TCGA-PAAD and GSE22780 datasets identified candidate hub genes. Prognostic relevance was assessed via Kaplan-Meier and ROC analyses. Functional experiments were performed in PANC-1 and BxPC-3 cells, including qRT-PCR, CCK-8 assay, Western blotting, Transwell assay, and apoptosis assay. Co-immunoprecipitation (Co-IP) was used to verify S100A14–S100A16 interaction. CHX chase and dual-luciferase assays were employed to assess protein stability and transcriptional activity.

**Results:**

*S100A14* was markedly upregulated in PC tissues and cell lines and identified as a key prognostic gene. Silencing *S100A14* suppressed EMT, proliferation, invasion, and migration, while reversing *S100A16*-mediated p53 inhibition and enhancing apoptosis. Mechanistically, Co-IP assay confirmed the protein interaction between *S100A14* and *S100A16*; *S100A14* stabilized *S100A16* protein through post-translational modification without transcriptional regulation; the *S100A14/S100A16* axis reduced *p53* protein stability and inhibited its transcriptional activity as well as the downstream *p21* expression. Critically, knockdown of *S100A14* abrogated the pro-metastatic phenotype of cancer cells.

**Conclusion:**

This study identifies *S100A14* promotes PC progression by stabilizing *S100A16* and suppressing the tumor-suppressive *p53/p21* pathway; knockdown of *S100A14* can reverse the above effects, restore *p53* function, and enhance cancer cell apoptosis. Targeting the *S100A14/S100A16/p53* regulatory axis could represent a promising therapeutic approach for PC.

## 1 Introduction

Pancreatic cancer (PC) is among the most deadly cancers, characterized by a steadily increasing incidence and mortality worldwide [1]. Notably, over 90% of PC cases are pancreatic ductal adenocarcinoma (PDAC), a highly aggressive subtype that drives the majority of its poor prognosis [2]. Epidemiological studies predict that over the next decade, the age-standardized mortality rate (ASMR) for PC will continue to rise significantly. By 2050, the global incidence is expected to reach 18.6 cases per 100,000 individuals [3]. Multiple risk factors contribute to the development of PC, including chronic pancreatitis, smoking, obesity, diabetes mellitus, and genetic predispositions [4,5]. Despite advances in surgical techniques, chemotherapy, and radiotherapy, the overall prognosis remains dismal due to the typically late-stage diagnosis and high metastatic potential [6]. Only approximately 12% of patients achieve a five-year survival, underscoring the limited survival benefits of current therapeutic strategies. The above issues show how urgently improved early detection techniques are needed to enhance therapeutic results.

S100A14, a calcium-binding protein that belongs to the S100 protein family, has been implicated in diverse roles across various cancer types [7]. This protein is known to modulate key signaling pathways such as EGFR, MAPK/ERK, tumor suppressor p53, and NF-κB [8]. *S100A14* plays a key role in a predictive model for PC based on seven immune-related genes, according to a new investigation. Its high expression is associated with higher M0 macrophage presence and lower CD8^+^ T cell infiltration [9]. Zhu et al. demonstrated that *S100A14* is overexpressed in PC, promoting tumor proliferation, migration, invasion, and chemoresistance, with elevated levels associated with advanced tumor stage and poor prognosis [10]. A previous study reported high expression levels of *S100A14* and *S100A16* in tumor tissues of breast cancer patients, suggesting that these proteins may promote breast cancer invasion by modulating the cytoskeleton [11]. Additionally, according to Tu et al., *S100A16* is upregulated in PC tissues and linked to poor patient outcomes, as well as immune cell infiltration, supported by a prognostic scoring system incorporating *S100A16* [12]. These findings underscore the importance of recognizing the molecular processes of *S100A14* and *S100A16* concerning PC, which may provide novel therapeutic targets.

Although several S100 proteins have been implicated in tumor progression, the regulatory hierarchy and mechanistic interplay among them in PC remain poorly defined. In particular, how specific S100 family members influence key tumor suppressors such as p53 is not fully understood. Previous studies suggest diverse interactions between S100 proteins and p53, yet the upstream modulators and post-translational mechanisms governing this axis are largely unexplored.

This study aims to delineate the S100A14/S100A16/p53 regulatory cascade, with an emphasis on post-translational control and functional consequences for cellular proliferation, invasion, and epithelial–mesenchymal transition (EMT). By elucidating this axis, we seek to uncover novel therapeutic targets that may restore p53 function and constrain the aggressive phenotype of PC.

## 2 Materials and Methods

### 2.1 Differential Expression Analysis and Overlapping Differentially Expressed Genes (DEGs) Identification

In this study, the Cancer Genome Atlas (TCGA)-Pancreatic adenocarcinoma (PAAD) dataset was downloaded from the ASSISTANT for Clinical Bioinformatics (https://www.aclbi.com), which included 179 tumor samples and 4 adjacent normal tissue samples. The GSE22780 dataset, comprising 8 PC samples and 8 normal pancreatic samples, was retrieved from the Gene Expression Omnibus (GEO, https://www.ncbi.nlm.nih.gov/gds/) database. Differential gene expression analysis between tumor and normal tissues was conducted independently for both datasets using the “limma” package in R software (version 4.2.0), this package is commonly used for differential expression analysis of microarray and RNA-seq data and can efficiently identify differentially expressed genes based on linear models. Only the log_2_ transformation was applied, as the GEO microarray data were already normalized by the original study. Genes with *p* < 0.05 and |log_2_FC| ≥ 0.38 (corresponding to fold change (FC) > 1.3 for upregulation or FC < 0.77) were considered differentially expressed, and no additional multiple-testing or batch-effect correction was applied, consistent with the exploratory nature of the analysis. The Bioinformatics & Evolutionary Genomics tool (https://bioinformatics.psb.ugent.be/webtools/Venn/) was utilized to perform Venn diagram analysis in order to find overlapping upregulated and downregulated DEGs between the GSE22780 and TCGA-PAAD datasets.

### 2.2 Protein-Protein Interaction (PPI) Network Construction and Hub Gene Identification

The STRING database (https://string-db.org/, version 12.0) was utilized to build a PPI network in order to investigate possible functional relationships between the overlapping upregulated and downregulated DEGs. The minimum required interaction score was set to 0.40 (medium confidence), as recommended by the STRING Consortium (ELIXIR core data resource, Switzerland). For additional investigation, the generated PPI network was loaded into the Cytoscape program (version 3.10.2; Cytoscape Consortium, San Diego, CA, USA). The cytoHubba plugin (version 0.1) was utilized to identify important hub genes, which ranks nodes based on network topology. Two independent algorithms, Maximum Clique Centrality (MCC) and EcCentricity, were employed to ensure the robustness of candidate hub gene selection. The ten genes that each algorithm evaluated highest were taken out and subjected to topological analysis to screen for candidate hub genes.

### 2.3 Prognostic Analysis and Receiver Operating Characteristic (ROC) Curve Evaluation

Utilizing Kaplan-Meier survival analysis and the Kaplan-Meier Plotter online tool (https://kmplot.com/analysis/), the prognostic significance of hub genes was evaluated. Overall survival (OS) information and gene expression data were obtained from the TCGA-PAAD cohort. Based on each gene’s median expression level, patients were divided into groups with high and low expression. The period of time between diagnosis and death from any cause was referred to as OS. To determine statistical significance, log-rank *p*-values and hazard ratios (HR) with 95% confidence interval (CI) were computed. To analyze the ROC curve, the R package “timeROC” was used to evaluate the predictive performance of each candidate gene. Area under the curve (AUC) values at 1-, 3-, and 5-year OS were calculated to compare the prognostic accuracy across genes.

### 2.4 Gene Expression Validation of Hub Genes

The Assistant for Clinical Bioinformatics (https://www.aclbi.com) provided the TCGA-PAAD dataset, which was utilized to analyze the expression levels of candidate hub genes in tumor and normal tissues. For consistency, we also evaluated candidate hub genes expression in the GSE22780 dataset. For both datasets, we analyzed gene expression levels using TPM normalization and assessed statistical differences between tumor and normal tissues using the Wilcoxon rank-sum test. Results are presented as boxplots generated using the Sangerbox platform (version 3.0, http://vip.sangerbox.com/home.html).

### 2.5 Cell Lines and Culture Conditions

Biovector NTCC (Beijing, China, http://www.biovector.net/) provided the human pancreatic ductal epithelial cell line HPDE6-C7, which was cultivated in MEM (Gibco, Thermo Fisher Scientific, Inc., Waltham, MA, USA; 11095080) with 10% FBS (Gibco, 10099141) added. We bought human PC cell lines from Procell (Wuhan, China, https://www.procell.com.cn/category/cytokines), which included Capan-1 (CL-0708), Capan-2 (CL-0709), PANC-1 (CL-0184), MiaPaca-2 (CL-0627), and BxPC-3 (CL-0042). All cell lines were authenticated using short tandem repeat (STR) profiling and confirmed to be free of mycoplasma contamination. The RPMI-1640 medium (Gibco, 31800022) supplemented with 10% FBS (Gibco, 10099141) and 1% penicillin-streptomycin (P/S; Gibco, 15140122) was used to cultivate all cancer cell lines. Every cell was kept in a humidified incubator with 5% CO_2_ at 37°C.

### 2.6 Cell Transfection and Treatment

PANC-1 and BxPC-3 cells were cultivated until they reached 70%–80% confluency after being seeded onto 6-well plates at a density of 5 × 10^5^ cells/well. Following the manufacturer’s instructions, Lipofectamine™ 2000 (Invitrogen, Thermo Fisher Scientific, Inc., Waltham, MA, USA; 11668019) was utilized for transfection. For knockdown experiments, 50 nM negative control siRNA (si-NC) or specific small interfering RNAs (siRNAs) targeting S100A14 (si-*S100A14*-1 and si-*S100A14*-2) were transfected into cells using 10 μL of Lipofectamine™ 2000 per well. The siRNA sequences were as follows: si-*S100A14*-1 forward, 5^′^-CCCAUCUCAUGCCGAGCAACU-3^′^, and reverse, 5^′^-AGUUGCUCGGCAUGAGAUGGG-3^′^; si-*S100A14*-2 forward, 5^′^-CCUCAUCAAGAACUUUCACCA-3^′^, and reverse, 5^′^-UGGUGAAAGUUCUUGAUGAGG-3^′^; and si-NC forward, 5^′^-UUCUCCGAACGUGUCACGU-3^′^, and reverse, 5^′^-ACGUGACACGUUCGGAGAATT-3^′^. For overexpression experiments, cells were transfected with 2 μg *S100A14* overexpression vector, *S100A16* overexpression vector, or empty vector (negative control). Cells were collected at 24 h post-transfection for subsequent analyses. Following transfection, cycloheximide (CHX, Sigma-Aldrich, St. Louis, MO, USA; C4859) at a concentration of 20 μg/mL to inhibit protein synthesis was administered to the cells. Cells were collected at the indicated time points (0, 30, 60, 90, and 120 min) post-CHX treatment for protein stability analysis.

### 2.7 Quantitative Real-Time PCR (qRT-PCR)

The TRIzol reagent (Tiangen, China; DP405) was applied to extract total RNA from cells in accordance with the manufacturer’s instructions. For cDNA synthesis, 1 μg of total RNA was reverse-transcribed using the PrimeScript RT Reagent Kit (Takara, Shiga, Japan; RR037A) in a 20 μL reaction system. qRT-PCR was performed utilizing a StepOnePlus Real-Time PCR System (Thermo Fisher Scientific, Inc., Waltham, MA, USA) with the SYBR Green PCR Master Mix (Vazyme, Nanjing, China). Each qPCR reaction was carried out in a 10 μL volume containing 5 μL SYBR Green Master Mix, 0.4 μL forward primer, 0.4 μL reverse primer, 1 μL cDNA template, and 3.2 μL nuclease-free water. The thermocycling procedure was as follows: 95°C for 30 s, followed by 40 cycles of 95°C for 10 s and 60°C for 30 s. Utilizing β-actin as the internal control, the 2^−ΔΔCt^ technique was utilized to quantify relative gene expression [13]. Table 1 contains primer sequences.

**Table 1:** Primer sequences for qRT-PCR

Target	Direction	Sequence (5^′^-3^′^)
*S100A14*	Forward	AGCGGCTGCCAACAGATCAT
*S100A14*	Reverse	TCTCAATGGCCCTCTCCACA
*S100A16*	Forward	ATCTGACTGTGGCTTGCTCA
*S100A16*	Reverse	GGCGGATCTGGATCTACTCT
*E-cadherin*	Forward	TGGACCGAGAGAGTTTCCCT
*E-cadherin*	Reverse	TCAAAATCCAAGCCCGTGGT
*N-cadherin*	Forward	TGGGAAATGGAAACTTGATGGC
*N-cadherin*	Reverse	AATCTGCAGGCTCACTGCTC
*Vimentin*	Forward	TCACCTGTGAAGTGGATGCC
*Vimentin*	Reverse	ACGAAGGTGACGAGCCATTT
*p53*	Forward	GTGACACGCTTCCCTGGATT
*p53*	Reverse	TCATCCATTGCTTGGGACGG
*β-actin*	Forward	AGCTCACCATGGATGATGATATCGC
*β-actin*	Reverse	CACATAGGAATCCTTCTGACCCAT

### 2.8 Western Blot (WB) Analysis

Protease and phosphatase inhibitor cocktails made of ice-cold RIPA buffer (Beyotime, Shanghai, China; P0013B) were utilized to lyse the cells. The BCA Protein Assay Kit (Beyotime, P0010) was applied to measure the protein concentrations. Proteins in equal quantities (20–30 μg per lane) were separated on 10% SDS-PAGE gels and then transferred utilizing a wet transfer technique (Bio-Rad) to PVDF membranes (Immobilon-P, Millipore, Billerica, MA, USA; IPVH00010). Membranes were blocked with 5% non-fat milk in Tris-buffered saline containing 0.1% Tween-20 (TBST) for 1 h at room temperature. Subsequently, membranes were incubated overnight at 4°C with the following primary antibodies diluted in 5% BSA/TBST: S100A14 (1:2000, ab233189), S100A16 (1:1000, ab240572), E-cadherin (1:1000, ab314063), N-cadherin (1:1000, ab245117), Vimentin (1:2000, ab92547), p53 (1:1000, ab26), p21 (1:1000, ab109199), β-actin (1:1000, ab8227) were primary antibodies (all purchased from Abcam, Cambridge, UK) diluted in 5% BSA/TBST were utilized to block the membranes for one hour at room temperature utilizing 5% non-fat milk in Tris-buffered saline with 0.1% Tween-20 (TBST) for an overnight incubation at 4°C. Membranes were then incubated with HRP-conjugated secondary antibodies (goat anti-rabbit, 1:2000, ab6721, Abcam) for one hour at room temperature following three rounds of TBST washing. Protein bands were seen employing an ECL detection method (Beyotime, P0018S). ImageJ software (version 2.0.0; National Institutes of Health, Bethesda, MD, USA) was utilized to take and analyze the images.

### 2.9 Cell Proliferation Assay

Cell proliferation was measured using Cell Counting Kit-8 (CCK-8, Dojindo, Kumamoto, Japan; CK04). Transfected cells were seeded into 96-well plates at a density of 5 × 10^3^ cells per well in 100 μL of complete medium. At 0, 12, 24, 48, and 72 h, each well received 10 μL of CCK-8 reagent, which was then incubated for 2 h. A Multiskan FC microplate reader (Thermo Fisher Scientific, Inc., Waltham, MA, USA) was employed to detect absorbance at 450 nm.

### 2.10 Transwell Assays

Transwell assays were applied to assess the ability of cells to invade and migrate. The upper chambers of Transwell inserts (8-μm pore size; Corning Costar, Corning, NY, USA) were filled with 5 × 10^4^ PANC-1 or BxPC-3 cells suspensions in serum-free media for the migration test, while the lower chambers contained 500 μL of serum-containing medium. The cells that remained in the upper chamber after 48 h of incubation at 37°C and did not migrate or invade were carefully removed utilizing a cotton swab. To see the nuclei, the cells on the membrane’s underside were fixed with 4% paraformaldehyde and labeled with DAPI (Beyotime, C1006). The stained cells were then counted and photographed in 5 randomly selected fields under an IX71 inverted microscope (Olympus, Tokyo, Japan; ×200). Prior to cell seeding, a basement membrane matrix (Matrigel; Corning, NY, USA; 354234; diluted 1:8 in serum-free medium) was applied to the Transwell inserts for the invasion experiment, and subsequent procedures were conducted similarly to the migration assay. Two types of experimental groups were included: (1) siRNA knockdown groups (si-NC, si-*S100A14*-1, si-*S100A14*-2); (2) overexpression + siRNA rescue groups (vector + si-NC, over-*S100A16* + si-NC, over-*S100A16* + si-*S100A14*-1).

### 2.11 Dual-Luciferase Reporter Assay

The luciferase assay was carried out in compliance with the guidelines provided by the manufacturer (Cignal p53 Reporter Assay Kit, SABiosciences, Frederick, MD, USA; CCS-004L). For assessing p53 transcriptional activity, pcDNA3.1-*S100A16* overexpressing plasmid–transfected cells (constructed by GeneChem, Shanghai, China) andtheir corresponding empty pcDNA3.1 vector controls, and/or *S100A14*-knockdown cells were plated at a density of 1 × 10^5^ cells per well in 12-well plates. By applying Lipofectamine 2000, these cells were transfected with a Renilla luciferase control plasmid and a p53-responsive firefly luciferase reporter plasmid (SABiosciences, Frederick, MD, USA). The transfection medium was swapped out for new complete media after 16 h. Luciferase activity was measured 72 h post-transfection following the aforementioned procedure using the Dual-Luciferase^®^ Reporter Assay System on a GloMax 20/20 luminometer (Promega, Madison, WI, USA), and firefly luciferase activity was normalized to Renilla luciferase activity.

### 2.12 Co-Immunoprecipitation (Co-IP) Assay

Cell lysates of PANC-1 and BxPC3 cells were prepared using lysis buffer supplemented with protease inhibitors. The protein concentration was determined with a BCA protein assay kit. For each Co-IP reaction, 200 μg of total protein was pre-incubated with Protein A/G PLUS-agarose beads (Santa Cruz Biotechnology, Dallas, TX, USA) at 4°C for 1 h with gentle rotation. The pre-incubated lysates were then incubated overnight at 4°C with gentle rotation in the presence of 2 μg of anti-S100A14 antibody (ab233189, Abcam) and 2 μg anti-S100A16 antibody (ab240572, Abcam). In parallel, normal rabbit IgG (Abcam, ab172730) was used as an IgG isotype negative control (IP:IgG) to confirm the specificity of the immunoprecipitation. After adding Protein A/G PLUS-agarose beads to the lysates, the mixture was further incubated at 4°C for 2 h. Following three washes with ice-cold lysis buffer, the beads were boiled in 2× SDS-PAGE loading buffer to elute the bound proteins. The eluted proteins were separated by SDS-PAGE and analyzed by WB using specific antibodies against S100A14, S100A16, and p53.

### 2.13 Flow Cytometry

PANC-1 cells were separated using trypsin-EDTA (Life Technologies, Beijing, China) for flow cytometry analysis and then cleaned with PBS. As directed by the manufacturer, cell apoptosis was detected using an Annexin V-FITC/PI Apoptosis Detection Kit (Beyotime, C1062M). Briefly, approximately 1 × 10^5^ cells were resuspended in binding buffer and incubated with Annexin V-FITC and propidium iodide (PI) for 15 min at room temperature in the dark, and dual staining channels (FITC and PI) were collected to distinguish between live, apoptotic, and necrotic cells. A CyFlow Cube 8 flow cytometer (Sysmex Partec GmbH, Görlitz, Germany) was used for the flow cytometry, and FlowJo software (version 10.8.1; FlowJo, LLC, Ashland, OR, USA) was used for data analysis to calculate the cell apoptosis rate.

### 2.14 Statistical Analysis

Utilizing the R language program (version 4.0.3), the statistical analysis was conducted. The data are shown as mean ± standard deviation (SD), with each experiment being conducted three times. The student’s *t*-test was used for comparisons between two groups, with this *t*-test was used for continuous values and to compare the means between the two groups. Tukey’s post hoc tests were utilized after a one-way ANOVA for comparisons involving several groups. Statistical significance was defined as a *p*-value of less than 0.05.

## 3 Results

### 3.1 Analysis of DEGs and Candidate Hub Genes Screening in PC-Related Datasets

**Figure 1:**
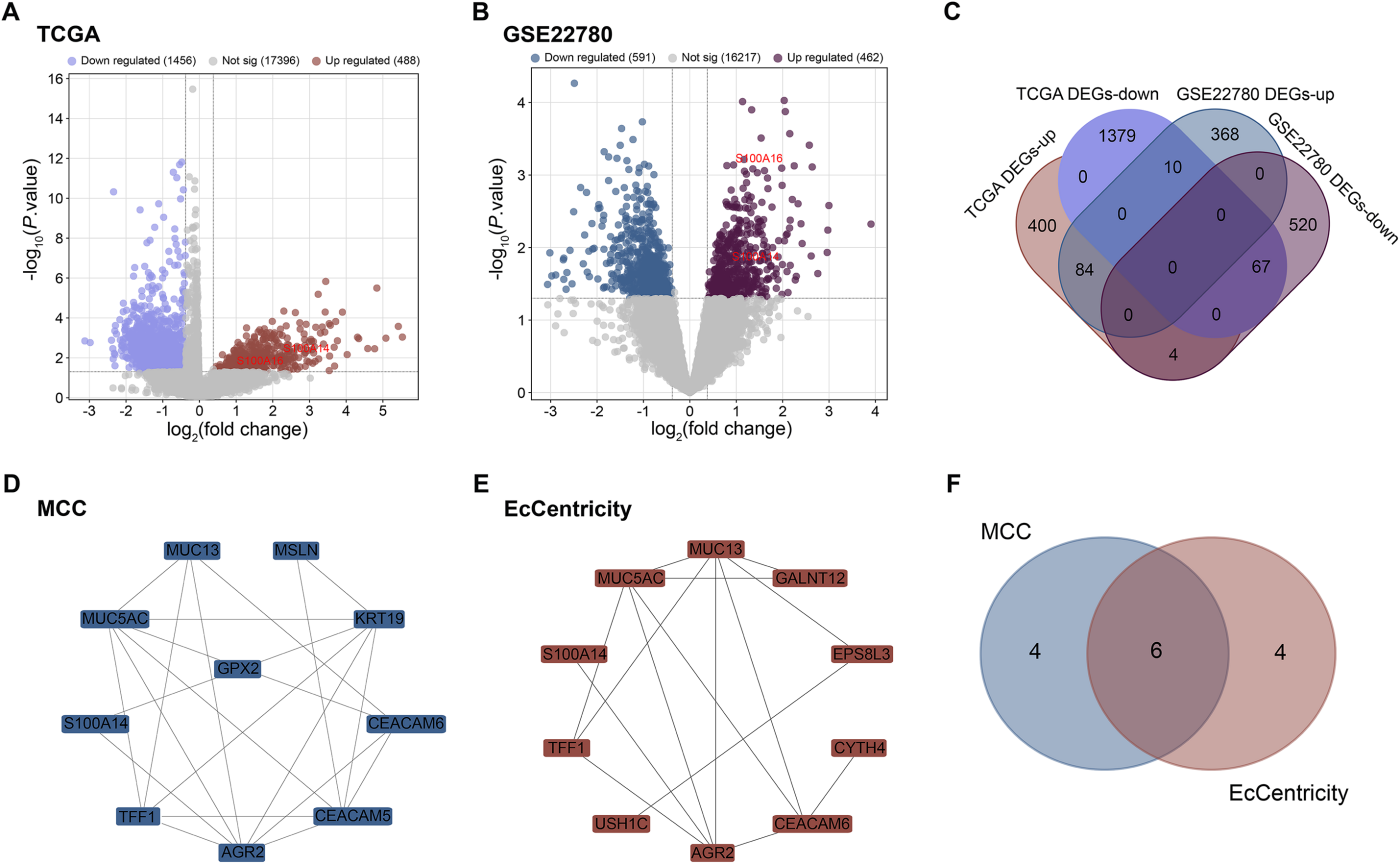
Identification of differentially expressed genes and hub genes in pancreatic cancer. (**A**) Volcano plot displaying DEGs from TCGA-PAAD dataset. Red dots represent significantly upregulated genes, blue dots indicate significantly downregulated genes. (**B**) Volcano plot showing DEGs from the GSE22780 dataset. Purple dots represent significantly upregulated genes, dark blue dots indicate significantly downregulated genes. (**C**) Venn diagram illustrating the number of overlapping upregulated and downregulated DEGs between TCGA-PAAD and GSE22780 datasets. (**D**) PPI network of DEGs with top 10 hub genes identified using the MCC. (**E**) PPI network showing top 10 hub genes identified by the EcCentricity algorithm. (**F**) Venn diagram depicting the intersection of hub genes derived from MCC and EcCentricity analyses. DEGs: Differentially Expressed Gene; PPI: Protein-Protein Interaction; TCGA-PAAD: The Cancer Genome Atlas-Pancreatic Adenocarcinoma; MCC: Maximal Clique Centrality

The TCGA-PAAD dataset yielded a total of 1456 downregulated and 488 upregulated DEGs (Fig. 1A), identified using the same criteria described in the Methods section (*p* < 0.05 and |log_2_FC| ≥ 0.38, corresponding to FC > 1.3 or FC < 0.77). In parallel, analysis of the GSE22780 dataset, using the identical thresholds, yielded 462 upregulated and 591 downregulated DEGs (Fig. 1B). Comparative analysis identified 84 overlapping upregulated DEGs and 67 overlapping downregulated DEGs between the two datasets (Fig. 1C). A PPI network was built in order to investigate the significance of these overlapping DEGs in more detail. Based on network connection measures, the top 10 hub genes were determined using the MCC and EcCentricity algorithms (Fig. 1D,E). As shown in Fig. 1F, subsequent topological analysis of the two algorithm-derived gene sets revealed six candidate hub genes (*MUC5AC*, *TFF1*, *AGR2*, *S100A14*, *CEACAM6*, *MUC13*).

### 3.2 Prognostic Evaluation and Expression Analysis of S100A14 as a Hub Gene in PC

**Figure 2:**
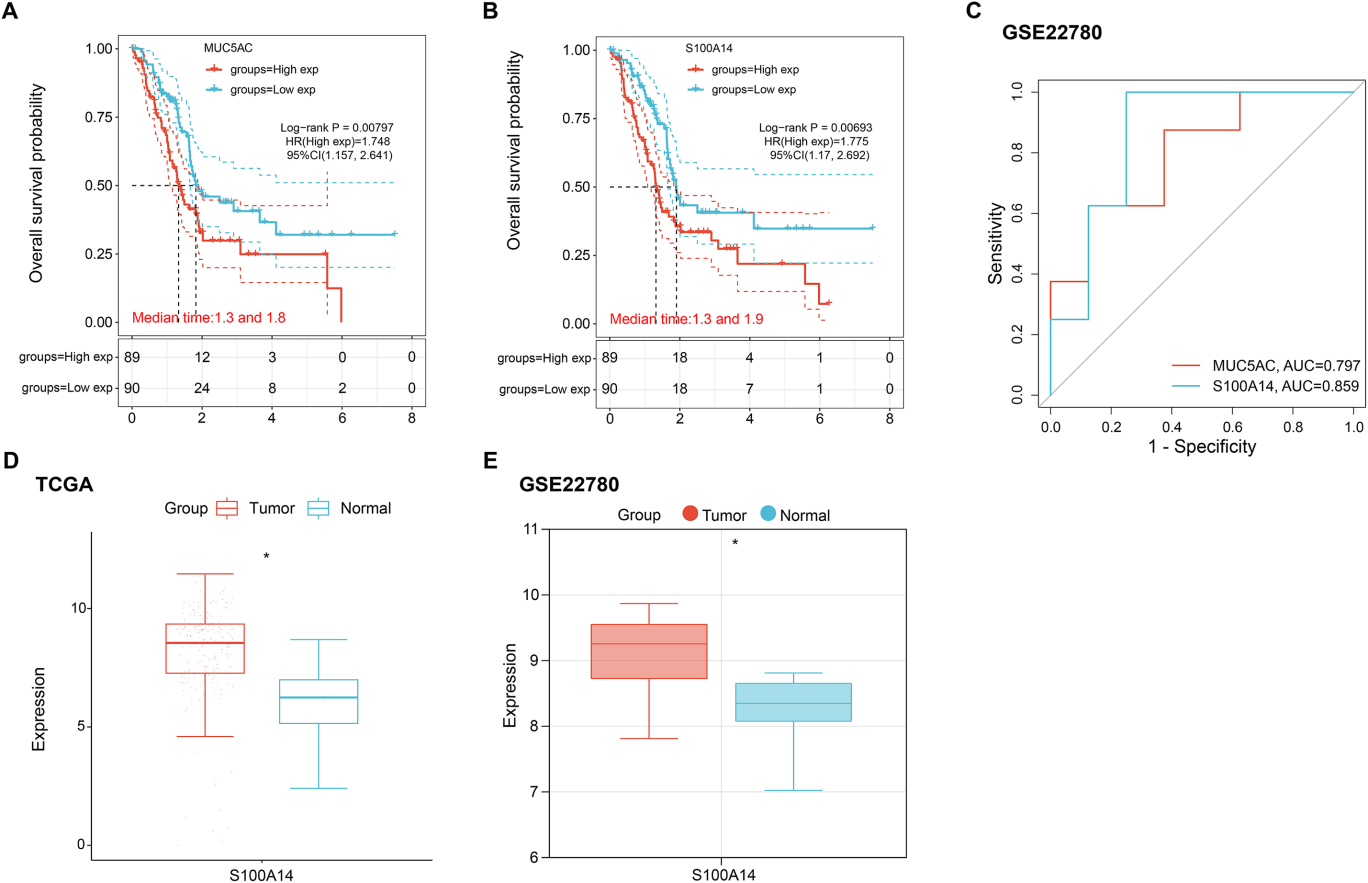
Prognostic and expression profiles of selected hub genes in pancreatic cancer. (**A**,**B**) Kaplan–Meier OS curves comparing high- and low-expression groups for *MUC5AC* (**A**) and *S100A14* (**B**). Log-rank tests were applied to assess statistical significance. (**C**) ROC curves assessing 1-year survival predictive accuracy for *MUC5AC* and *S100A14*; the area under the curve (AUC) values are indicated. (**D**,**E**) Boxplots showing *S100A14* mRNA expression levels in pancreatic tumor and normal tissue samples from TCGA-PAAD (**D**) and GSE22780 (**E**) datasets. OS: Overall Survival; TCGA-PAAD: The Cancer Genome Atlas-Pancreatic Adenocarcinoma; ROC: Receiver Operating Characteristic. *p < 0.05

**Figure A1:**
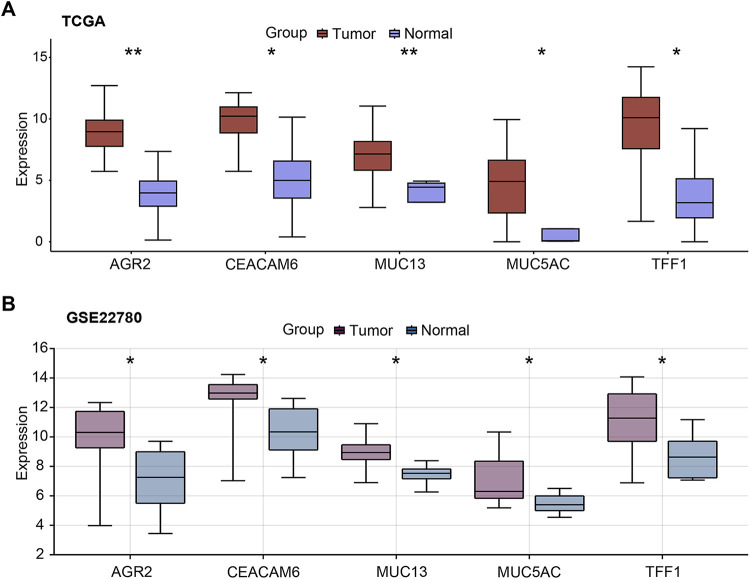
Expression profiles of hub genes in pancreatic cancer. (**A**,**B**) Boxplots showing *AGR2*, *CEACAM6*, *MUC13*, *MUC5AC*, and *TFF1* mRNA expression levels in pancreatic tumor and normal tissue samples from TCGA-PAAD (**A**) and GSE22780 (**B**) datasets. TCGA-PAAD: The Cancer Genome Atlas-Pancreatic Adenocarcinoma. *p < 0.05, **p < 0.01

Survival analysis of the six candidate hub genes revealed that only *MUC5AC* (Log-rank *p* = 0.00797) and *S100A14* (Log-rank *p* = 0.00693) exhibited statistically significant associations with OS. Subgroup stratification analysis further demonstrated that high-risk patients groups stratified by *MUC5AC* and *S100A14* expression levels showed significantly shortened OS compared to their low-risk counterparts (Fig. 2A,B). ROC curve analysis comparing the predictive efficacy of *MUC5AC* and *S100A14* revealed that *S100A14* demonstrated a significantly higher area under the curve (AUC = 0.859), indicating superior discriminative power for outcome prediction (Fig. 2C). Based on these findings, *S100A14* was selected as the hub gene for further investigation. In addition to S100A14, the remaining five candidate hub genes (*AGR2*, *CEACAM6*, *MUC13*, *MUC5AC*, and *TFF1*) also underwent expression validation. As shown in Fig. A1A,B, all five genes displayed significantly elevated expression in pancreatic tumor tissues compared with normal pancreatic tissues in both the TCGA-PAAD and GSE22780 datasets, supporting their potential involvement in pancreatic cancer pathogenesis. Finally, expression analysis of *S100A14* in the TCGA-PAAD and GSE22780 datasets showed upregulation in tumor tissues compared to normal controls (Fig. 2D,E).

### 3.3 Elevated Expression of S100A14 in PC Cell Lines and Its Functional Role in Regulating Cell Proliferation

**Figure 3:**
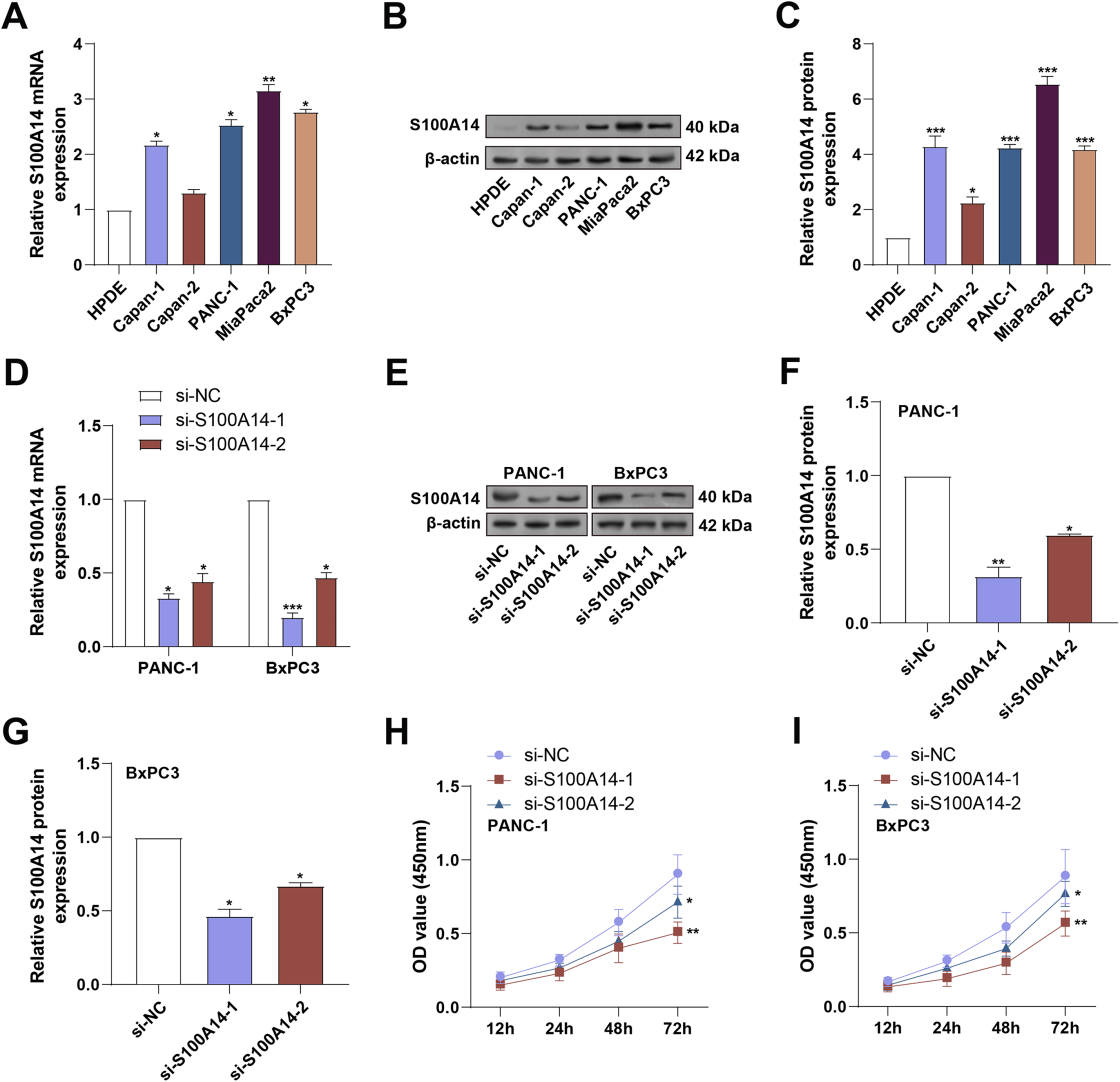
Expression validation and knockdown efficiency of *S100A14* in pancreatic cancer cell lines. (**A**) qRT-PCR analysis of *S100A14* mRNA expression in normal pancreatic ductal epithelial (HPDE) cells and PC cell lines (Capan-1, Capan-2, PANC-1, MiaPaCa-2, BxPC3). (**B**) WB analysis of *S100A14* protein expression in HPDE, Capan-1, Capan-2, PANC-1, MiaPaCa-2, BxPC3. (**C**) Quantification of the relative S100A14 protein levels corresponding to panel B. Bars represent normalized densitometry values. (**D**,**E**) Knockdown efficiency of two independent siRNAs targeting S100A14 (si-*S100A14*-1 and si-*S100A14*-2) in PANC-1 and BxPC3 cells was confirmed by qRT-PCR (**D**) and WB (**E**). (**F**,**G**) Quantification of WB results showing reduced S100A14 protein levels in PANC-1 (**F**) and BxPC3 (**G**) cells following siRNA transfection. (**H**,**I**) CCK-8 assay results showing relative proliferative capacity of PANC-1 (**H**) and BxPC3 (**I**) cells following *S100A14* knockdown; absorbance measured at 450 nm. qRT-PCR: Quantitative Real-Time Polymerase Chain Reaction; WB: Western Blotting; CCK-8: Cell Counting Kit-8. *p < 0.05, **p < 0.01, ***p < 0.001

To validate the expression of *S100A14*, qRT-PCR and WB analyses were conducted in normal HPDE cells and a panel of PC cell lines, including Capan-1, MiaPaCa-2, Capan-2, PANC-1, and BxPC3. As shown in Fig. 3A–C, *S100A14* was markedly upregulated in all PC cell lines relative to HPDE cells, with MiaPaCa-2 exhibiting the highest expression. PANC-1 and BxPC-3 showed moderately high, but not saturated, S100A14 expression, making them suitable for both gain and loss-of-function manipulation; therefore, these two cell lines were selected for subsequent functional experiments. Following transfection with si-*S100A14*-1 and si-*S100A14*-2, a significant downregulation of *S100A14* expression was observed in both cell lines, as confirmed by qRT-PCR and WB assays (Fig. 3D–G). Using the CCK-8 test, the effect of *S100A14* knockdown on cell proliferation was evaluated. *S100A14* knockdown significantly decreased the ability of PANC-1 and BxPC3 cells to proliferate (Fig. 3H,I).

### 3.4 S100A14 Knockdown Suppresses Migration, Invasion, and EMT in PC Cells

**Figure 4:**
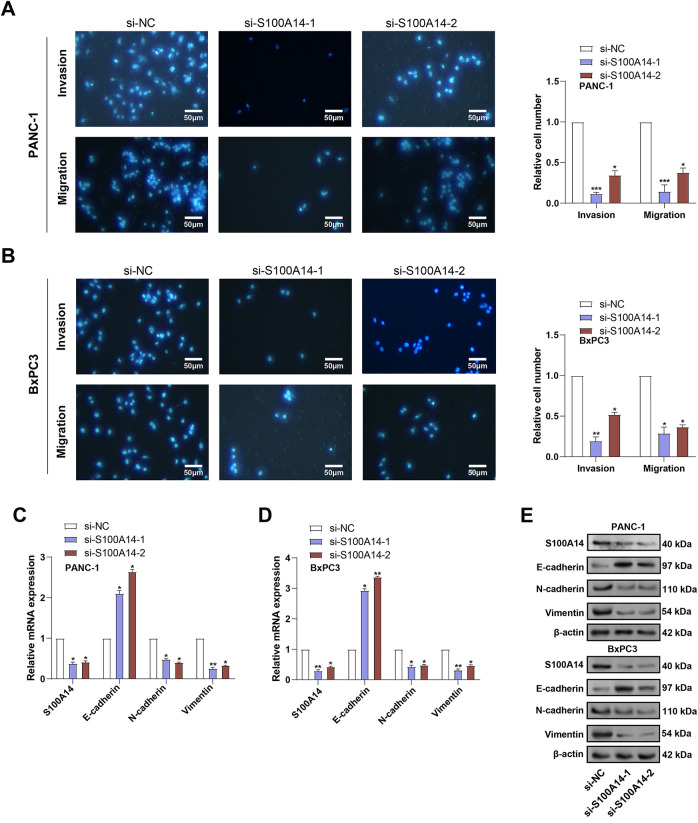

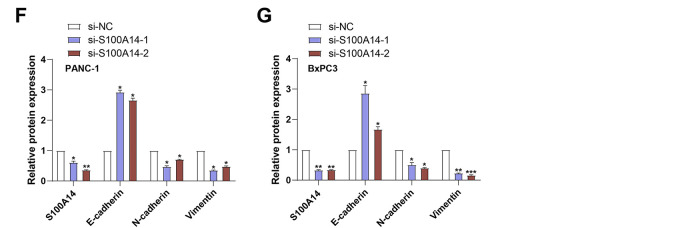
Impact of *S100A14* knockdown on migration, invasion, and EMT markers in pancreatic cancer cells. (**A**,**B**) Transwell assays quantifying migrated and invaded PANC-1 (**A**) and BxPC3 (**B**) cells with or without *S100A14* silencing. Magnification: 200×, scale bar = 50 μm. (**C**) Relative mRNA expression levels of S100A14, E-cadherin, N-cadherin, and Vimentin in PANC-1 cells after transfection with si-NC, si-S100A14-1, or si-S100A14-2, as determined by qRT-PCR. (**D**) Relative mRNA expression of the same genes in BxPC-3 cells treated as in (**C**). (**E**) Representative Western blot images showing S100A14 and EMT-related proteins (E-cadherin, N-cadherin, and Vimentin) in PANC-1 and BxPC-3 cells following S100A14 knockdown; β-actin served as the loading control. (**F**) Densitometric analysis of protein expression in PANC-1 cells corresponding to the Western blots in (**E**). (**G**) Densitometric analysis of protein expression in BxPC-3 cells corresponding to the Western blots in (**E**). EMT: epithelial-mesenchymal transition. *p < 0.05, **p < 0.01, ***p < 0.001

To investigate the role of *S100A14* in PC cell motility, Transwell assays were conducted following *S100A14* knockdown in PANC-1 and BxPC3 cells. As shown in Fig. 4A,B, silencing *S100A14* significantly impaired both invasive and migratory capacities of the two cell lines, indicating its promotive function in PC cell dissemination. Subsequently, applying qRT-PCR and WB analysis, the expression of markers linked to EMT was investigated. E-cadherin was significantly upregulated when *S100A14* was knocked down, although N-cadherin, Vimentin, and *S100A14* itself were all expressed less in both cell types (Fig. 4C–G). These findings suggest that *S100A14* may facilitate EMT in PC cells, thereby contributing to their migratory and invasive behavior.

### 3.5 S100A14 Regulates S100A16 Protein but Not mRNA Expression in PC Cells

**Figure 5:**
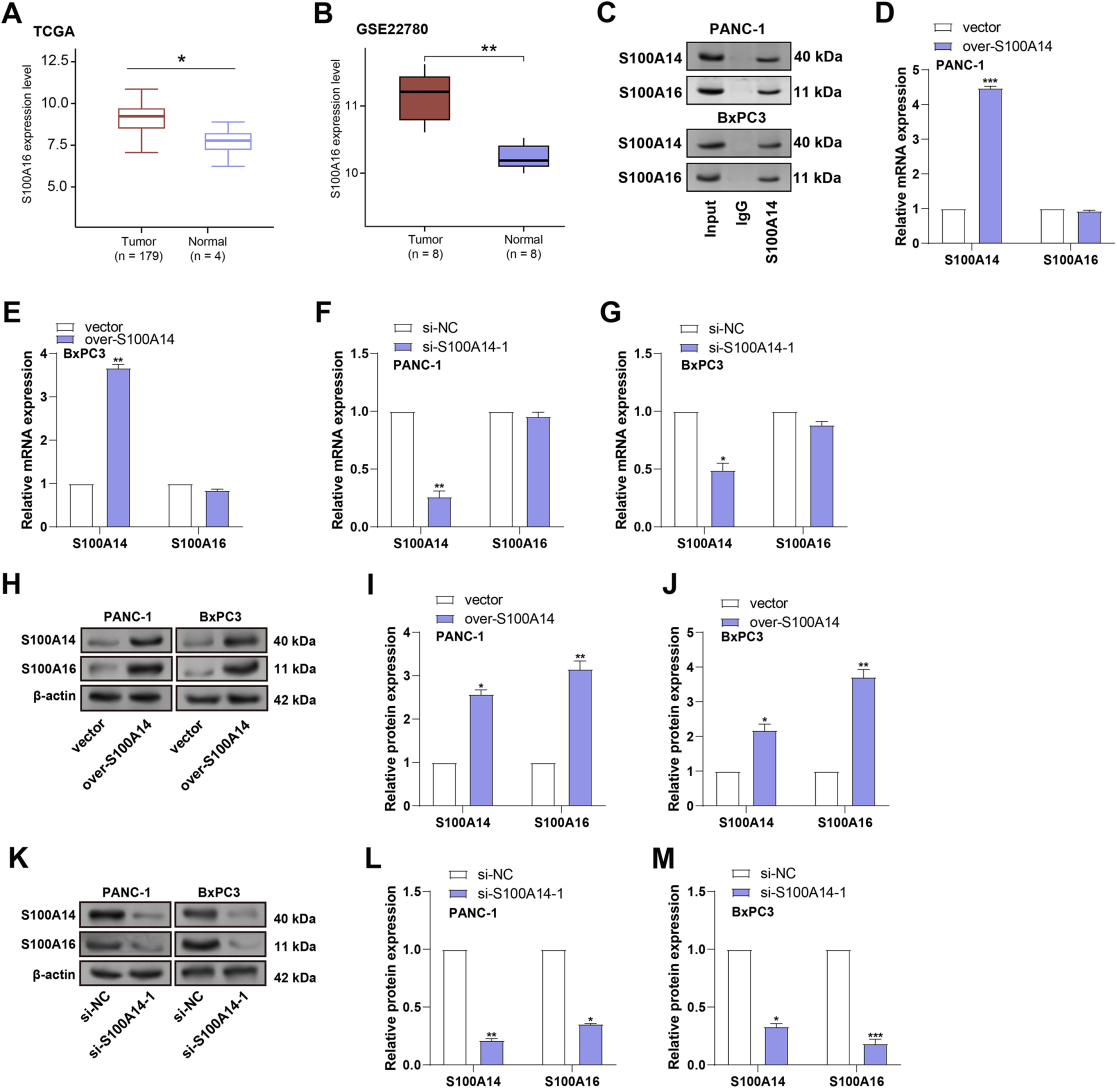
Post-transcriptional regulation of *S100A16* protein expression by *S100A14* in pancreatic cancer cells. (**A**) Boxplot representing *S100A16* expression in TCGA-PAAD tumor vs. normal tissues. (**B**) Boxplot representing *S100A16* expression in GSE22780 tumor vs. normal tissues. (**C**) Co-IP assay verifies the direct interaction between S100A14 and S100A16 in PANC-1 and BxPC3 cells. (**D**,**E**) qRT-PCR analysis of *S100A14* and *S100A16* mRNA expression after *S100A14* overexpression in PANC-1 (**D**) and BxPC3 (**E**) cells. (**F**,**G**) qRT-PCR assessment of *S100A14* and *S100A16* mRNA levels following *S100A14* knockdown in PANC-1 (**F**) and BxPC3 (**G**) cells. (**H**) Representative WB images showing that *S100A14* overexpression markedly increased S100A16 protein levels in both PANC-1 and BxPC-3 cells, while β-actin served as a loading control. (**I**) Quantification of Western blots showing increased S100A14 and S100A16 protein expression in PANC-1 cells upon *S100A14* overexpression. (**J**) Quantitative analysis showing that *S100A14* overexpression significantly upregulated S100A16 protein expression in BxPC-3 cells. (**K**) WB analysis showing that *S100A14* knockdown decreased S100A16 protein expression in both PANC-1 and BxPC-3 cells. (**L**) Quantification of Western blots showing that si-*S100A4*-1 significantly reduced S100A14 and S100A16 protein levels in PANC-1 cells. (**M**) Quantitative analysis showing that *S100A14* knockdown markedly decreased S100A16 protein expression in BxPC-3 cells. Co-IP: Co-immunoprecipitation. *p < 0.05, **p < 0.01, ***p < 0.001

A previous study has identified *S100A14* and *S100A16* as interacting partners, with evidence suggesting that *S100A14* regulates S100A16 protein expression through a non-transcriptional mechanism [14]. Both proteins undergo time-dependent degradation that appears independent of classical proteasomal or lysosomal pathways. To assess the clinical relevance of *S100A16* in PC, expression analysis using the TCGA-PAAD and GSE22780 datasets was performed. It was discovered that tumor tissues have much higher levels of *S100A16* than normal groups (Fig. 5A,B). To verify the direct interaction between S100A14 and S100A16, we performed co-immunoprecipitation (Co-IP) assays in PANC-1 and BxPC3 cells. In PANC-1 cells, following immunoprecipitation with an anti-S100A16 antibody, a co-precipitated band of S100A14 was detected. In BxPC3 cells, immunoprecipitation with an anti-S100A14 antibody also resulted in the detection of a co-precipitated band of S100A16 (Fig. 5C). These results demonstrate that S100A14 and S100A16 directly interact within cells. To investigate the regulatory relationship between *S100A14* and *S100A16*, qRT-PCR was performed to assess the mRNA levels of both genes in PANC-1 and BxPC3 cells following *S100A14* overexpression or knockdown. As expected, overexpression of *S100A14* markedly increased *S100A14* mRNA expression, while knockdown significantly decreased its mRNA levels. Interestingly, modulation of *S100A14* expression did not affect the mRNA expression of *S100A16* (Fig. 5D–G). Further, Under the same circumstances, WB analysis was performed to assess the protein expression levels of S100A14 and S100A16. Notably, in PANC-1 and BxPC3 cells, overexpression of *S100A14* resulted in a large rise in the levels of both S100A14 and S100A16 proteins (Fig. 5H–J), whereas knockdown of *S100A14* led to a marked decrease in both proteins (Fig. 5K–M). These experimental results show that *S100A14* and *S100A16* have a direct interaction in cells. Meanwhile, the mRNA level of *S100A16* remains stable, whereas its protein level changes with the expression of *S100A14*. Notably, this finding is consistent with previous research conclusions: *S100A14* and *S100A16* are directly interacting proteins; the former can regulate the protein expression of the latter through a non-transcriptional mechanism; and the degradation processes of both proteins are independent of the classical proteasomal or lysosomal pathways. Based on this, we hypothesize that in pancreatic cancer cells, *S100A14* most likely achieves specific regulation of *S100A16* protein levels by directly binding to *S100A16* protein, and this regulatory process is not associated with the transcriptional process of *S100A16*.

### 3.6 S100A14 Unidirectionally Regulates the Expression of S100A16

**Figure 6:**
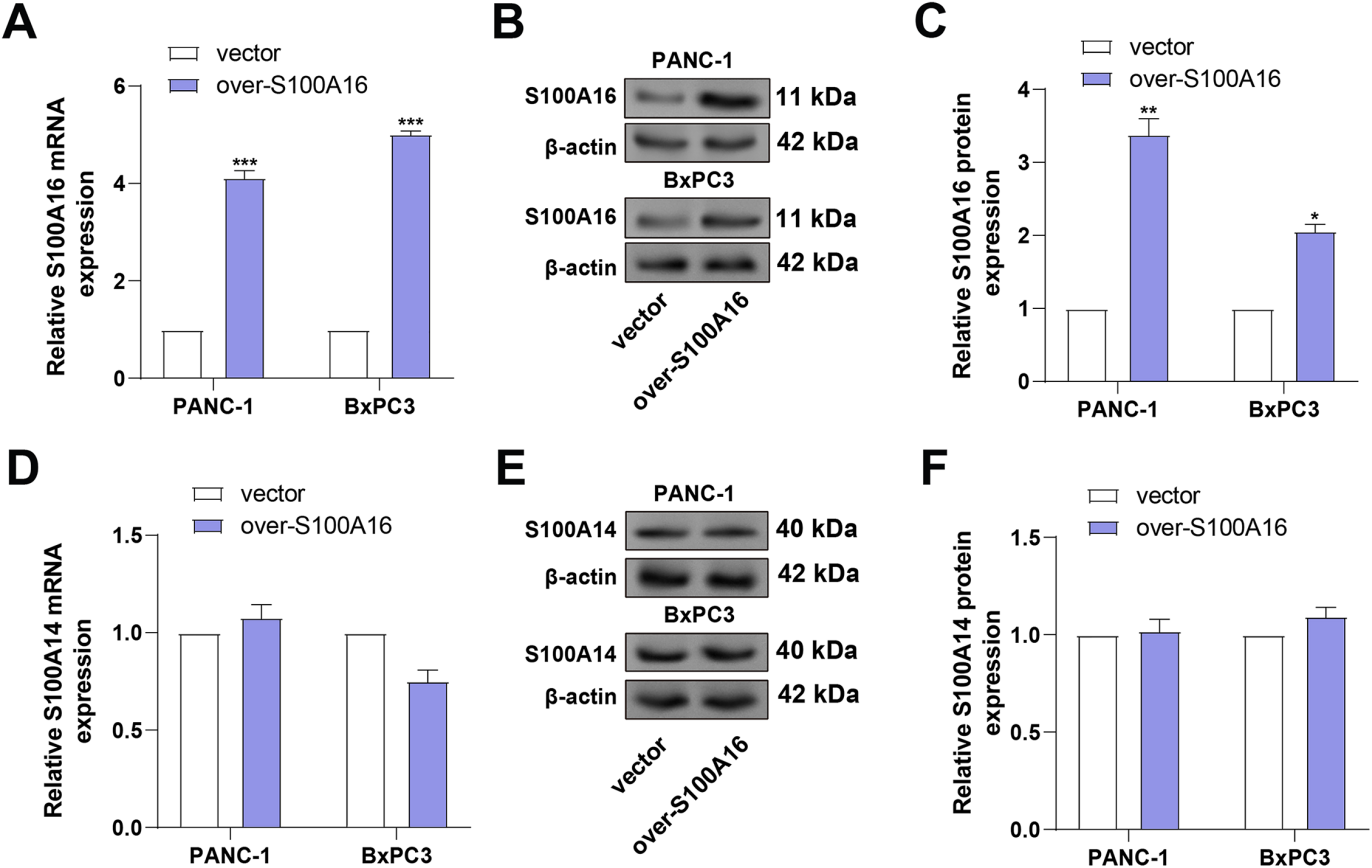
Validation of *S100A16* overexpression and evaluation of its effect on *S100A14* expression. (**A**) qRT-PCR quantification of *S100A16* mRNA levels in PANC-1 and BxPC3 cells after transfection with *S100A16* overexpression plasmids. (**B**,**C**) WB analysis confirming increased *S100A16* protein expression post-transfection. (**D**) qRT-PCR analysis of *S100A14* mRNA expression in PANC-1 and BxPC3 cells following *S100A16* overexpression. (**E**) WB showing *S100A14* protein levels after *S100A16* overexpression in PANC-1 and BxPC3 cell lines. (**F**) Quantification of S100A14 protein expression corresponding to (**E**), showing that S100A14 protein levels were not significantly altered by *S100A16* overexpression. *p < 0.05, **p < 0.01, ***p < 0.001

To further evaluate the regulatory relationship between *S100A14* and *S100A16*, PANC-1 and BxPC3 cells were transfected with *S100A16*-overexpressing plasmids. Following transfection, *S100A16* was shown to be markedly increased at both the mRNA and protein levels by qRT-PCR and WB studies (Fig. 6A–C), validating the transfection efficiency. Subsequently, the impact of *S100A16* overexpression on *S100A14* expression was assessed. Neither the mRNA nor protein levels of *S100A14* were altered in response to elevated *S100A16* expression in either cell line (Fig. 6D–F). These results, in conjunction with earlier findings, support a unidirectional regulatory relationship, whereby *S100A14* modulates *S100A16* protein expression without reciprocal regulation.

### 3.7 S100A14 Knockdown Abrogates S100A16-Induced Migration, Invasion, and EMT in PC Cells

**Figure 7:**
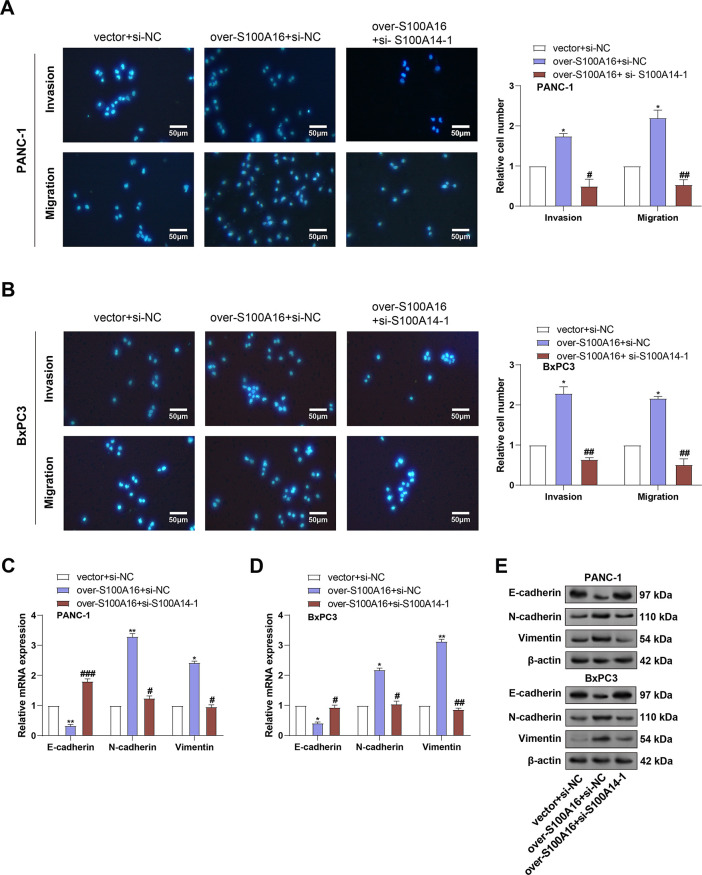

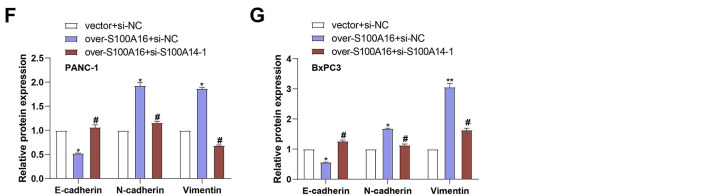
Effects of *S100A16* overexpression and combined *S100A14* knockdown on PC cell migration, invasion, and EMT marker expression. (**A**,**B**) Transwell assays assessing migration and invasion of PANC-1 (**A**) and BxPC3 (**B**) cells under *S100A16* overexpression alone or combined with *S100A14* knockdown. Magnification: 200×, scale bar = 50 μm. (**C**) qRT-PCR quantification of *E-cadherin*, *N-cadherin*, and *Vimentin* mRNA expression in PANC-1 cells. (**D**) Quantification of *E-cadherin*, *N-cadherin*, and *Vimentin* mRNA expression in BxPC3 cells. (**E**) WB analysis of E-cadherin, N-cadherin, and Vimentin protein levels under the indicated conditions. (**F**) Densitometric quantification of E-cadherin, N-cadherin, and Vimentin protein levels in PANC-1 cells corresponding to the Western blots in (**E**). (**G**) Densitometric quantification of E-cadherin, N-cadherin, and Vimentin protein levels in BxPC-3 cells corresponding to the Western blots in (**E**). qRT-PCR: Quantitative real-time polymerase chain reaction; WB: Western blotting. *p < 0.05 or **p < 0.01 vs. vector+si-NC group, ^#^*p* < 0.05 or ^##^*p* < 0.01 or ^###^*p* < 0.001 vs. over-*S100A16*+si-NC group

To clarify *S100A14* and *S100A16* contribute to PC cell motility, Transwell assays were performed in PANC-1 and BxPC3 cells following *S100A16* overexpression or combined *S100A16* overexpression and *S100A14* knockdown. As shown in Fig. 7A,B, overexpression of *S100A16* markedly enhanced both migratory and invasive capacities in both cell lines. Notably, these pro-metastatic effects were reversed upon concurrent knockdown of *S100A14*. Subsequently, EMT marker expression was evaluated using qRT-PCR and WB analysis. Vimentin and N-cadherin levels rose in response to overexpression of *S100A16*, but E-cadherin expression decreased, indicative of EMT induction. However, co-silencing of *S100A14* restored E-cadherin level and suppressed Vimentin and N-cadherin (Fig. 7C–G).

### 3.8 S100A14 Overexpression Reduces p53 Protein Expression by Inhibiting Its Transcriptional Activity and Promoting Degradation

**Figure 8:**
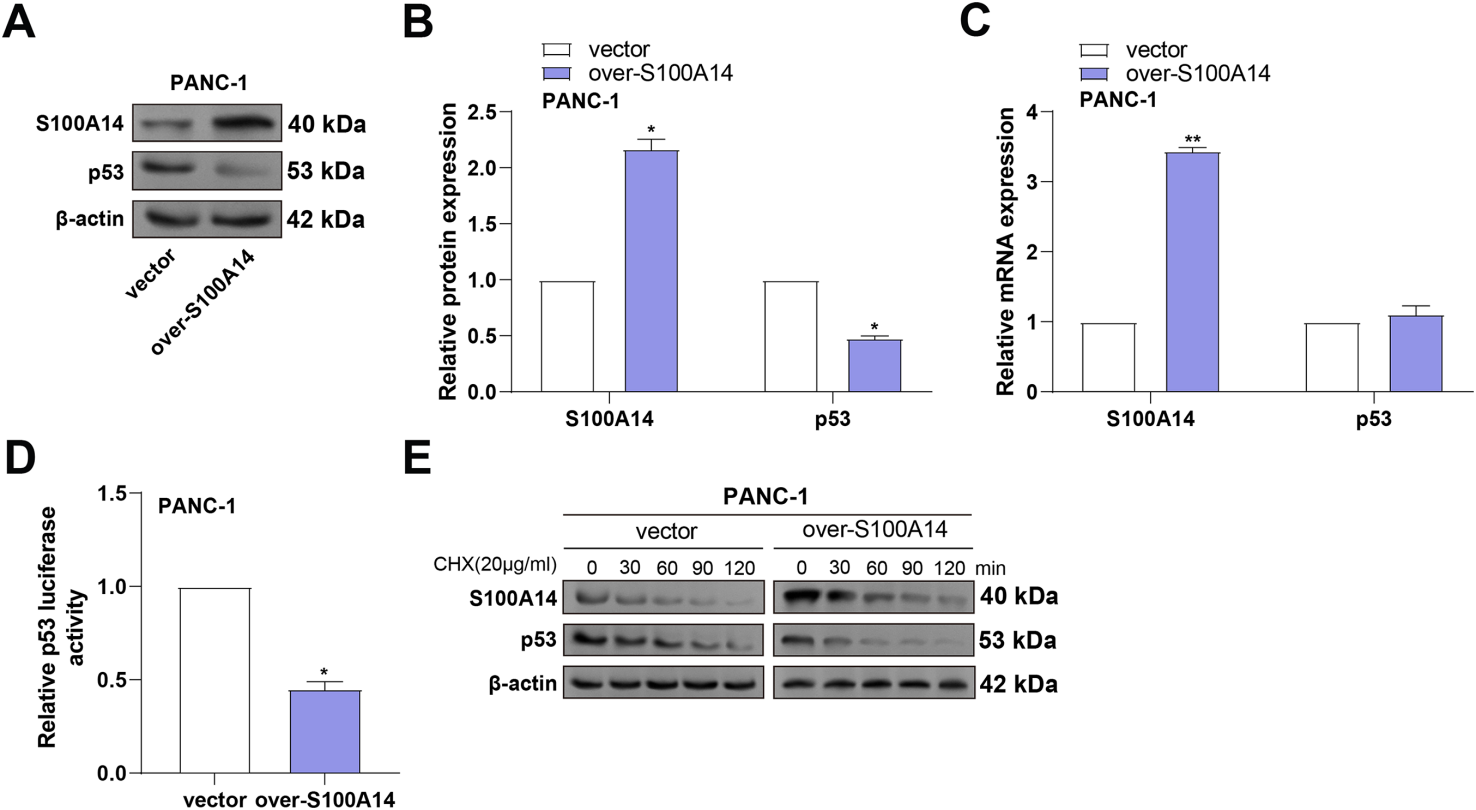
*S100A14* overexpression suppresses p53 protein levels by reducing protein stability in PANC-1 cells. (**A**) WB analysis showing increased S100A14 and decreased p53 protein expression after *S100A14* overexpression. (**B**) Quantification of S100A14 and p53 protein expression normalized to β-actin. (**C**) qRT-PCR measurement of *S100A14* and *p53* mRNA levels in control and *S100A14*-overexpressing cells. (**D**) Dual-luciferase reporter assay measuring transcriptional activity of p53 using a p53-responsive luciferase plasmid; relative luciferase units normalized to control. (**E**) Western blot analysis of p53 protein stability in control and *S100A14*-overexpressing PANC-1 cells treated with cycloheximide (CHX, 20 μg/mL) for the indicated time points (0–120 min). *p < 0.05, **p < 0.01

*p53* is a critical tumor suppressor that maintains genomic stability by preventing the proliferation of damaged cells, thereby inhibiting tumorigenesis [15]. In addition, studies have found that *p53* interacts with multiple members of the S100 protein family, which can regulate the transcriptional activity and biological functions of *p53* in various ways [16,17]. To examine *S100A14’*s regulatory role in p53 expression, WB analysis was performed in PANC-1 cells following *S100A14* overexpression. As shown in Fig. 8A,B, *S100A14* overexpression led to a marked increase in its own protein level, accompanied by a notable reduction in p53 protein expression. To ascertain whether transcriptional regulation was involved, qRT-PCR was conducted. While *S100A14* mRNA expression was significantly upregulated, no significant change in *p53* mRNA levels was observed (Fig. 8C), suggesting a post-transcriptional regulatory mechanism. As shown by dual-luciferase reporter experiments, overexpression of *S100A14* markedly reduced p53 transcriptional activity (Fig. 8D), indicating suppression of p53 function. To further explore whether S100A14 affects p53 protein stability, cycloheximide (CHX) chase assays were conducted. As shown in Fig. 8E, *S100A14* overexpression accelerated the degradation of p53 protein over time in contrast to the control group. These results suggest that *S100A14* suppresses p53 expression primarily through inhibition of its transcriptional activity and promotion of proteasome-mediated degradation.

### 3.9 S100A14 Knockdown Reverses S100A16-Mediated Suppression of the p53/p21 Pathway in PC Cells

**Figure 9:**
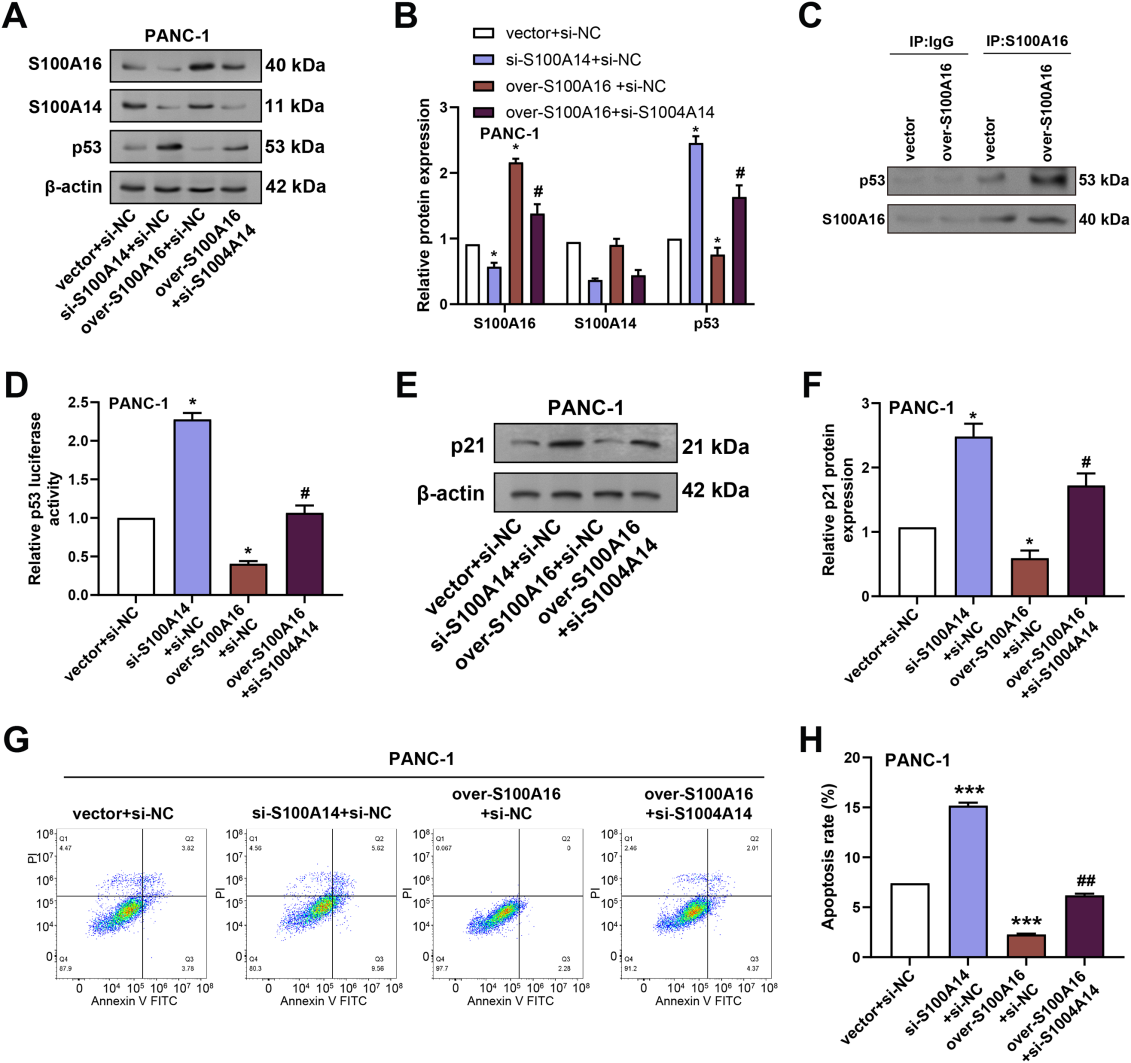
Modulation of p53 and p21 expression by *S100A16* overexpression and *S100A14* knockdown in PC cells. (**A**) WB analysis of S100A14, S100A16, and p53 protein levels in PANC-1 cells transfected with S100A14 knockdown plasmid alone, S100A16 overexpression plasmid alone, or in combination with S100A14 knockdown. (**B**) Quantification of S100A16, S100A14, and p53 protein expression normalized to β-actin. (**C**) Co-IP assay confirming physical interaction between *S100A16* and p53 proteins. (**D**) Luciferase reporter assay evaluating p53 transcriptional activity in cells with S100A14 knockdown, S100A16 overexpression, or S100A16 overexpression combined with S100A14 knockdown. (**E**) WB analysis of p21 protein expression following the indicated treatments. (**F**) Quantification of p21 protein expression normalized to β-actin. (**G**) Flow cytometry was used to detect the apoptosis of PANC-1 cells in different treatment groups. (**H**) Statistical analysis of apoptosis rates in PANC-1 cells. Co-IP: Co-immunoprecipitation. *p < 0.05 or ***p < 0.001 vs. vector+si-NC group, ^#^*p* < 0.05 or ^##^*p* < 0.05 vs. over-*S100A16*+si-NC group

To determine whether S100A14 modulates the regulatory effects of S100A16 on the p53 signaling pathway and cell apoptosis, we performed rescue experiments in PANC-1 cells. WB analysis was conducted to assess the effects of *S100A16* overexpression and/or *S100A14* knockdown on the protein levels of *S100A14*, *S100A16*, and *p53* in PANC-1 cells. Overexpression of *S100A16* significantly increased *S100A16* protein expression while reducing *p53* protein levels without altering *S100A14* expression. Conversely, combined *S100A16* overexpression and *S100A14* knockdown resulted in decreased protein levels of both *S100A16* and *S100A14*, accompanied by an increase in *p53* protein expression (Fig. 9A,B). Co-immunoprecipitation assays confirmed a direct interaction between *S100A16* and *p53* in PANC-1 cells (Fig. 9C). To investigate the functional consequence of this interaction, *p53* transcriptional activity was evaluated using a *p53*-responsive luciferase reporter assay. Knockdown of *S100A14* significantly promoted *p53*-driven luciferase activity, while *S100A16* overexpression significantly suppressed *p53*-driven luciferase activity, and this suppression was restored by concurrent *S100A14* knockdown (Fig. 9D). Further WB analysis revealed that overexpression of *S100A16* decreased the expression of *p21*, a canonical downstream target of *p53*, and this reduction was reversed when *S100A14* was simultaneously knocked down (Fig. 9E,F). Meanwhile, cell apoptosis was detected by flow cytometry: the apoptosis rate was significantly increased in the si-*S100A14*+si-NC group, decreased in the over-*S100A16*+si-NC group, and the over-*S100A16*+si-*S100A14* group reversed the apoptosis inhibition mediated by over-*S100A16* (Fig. 9G,H). In conclusion, *S100A14* is involved in *S100A16*-mediated regulation of the *p53/p21* tumor suppressor axis and cell apoptosis. Knockdown of *S100A14* can alleviate the inhibitory effect of *S100A16* on the *p53/p21* pathway and promote cancer cell apoptosis.

## 4 Discussion

PC is frequently diagnosed at advanced stages, which severely limits therapeutic options and contributes to its poor survival rates [18]. Increasing evidence underscores the significant clinical and prognostic relevance of the S100 protein family in pancreatic ductal adenocarcinoma (PDAC) [19,20]. One example is the thorough examination of six members of the S100A family (*S100A2*, *S100A4*, *S100A6*, *S100A10*, *S100A14*, and *S100A16*) by Li et al., revealing their marked overexpression and reduced methylation levels in PDAC tissues, with elevated expression correlating negatively with patient survival [21]. Similarly, High expression of various S100A members, such as *S100A14* and *S100A16*, has been linked by Zhuang et al. to advanced tumor stage, poor histological grade, and an adverse prognosis, highlighting their roles in tumor progression and immune regulation [22]. Bioinformatics analysis in this study revealed that only *MUC5AC* and *S100A14* were significantly associated with overall survival. Moreover, *S100A14* demonstrated excellent predictive performance with AUC = 0.859 and was consistently upregulated in tumor tissues across two pancreatic cancer-related datasets.

The role of *S100A14* has been explored in various malignancies with diverse outcomes. For instance, Jiang et al. reported that Prostate cancer tissues and cell lines have downregulated expression of *S100A14*, which slows tumor growth by upregulating FAT1 and triggering the Hippo signaling pathway. This activation promotes apoptosis while inhibiting cell migration, proliferation, and EMT [23]. Zhang et al. further showed that *ZHX2* inhibits thyroid carcinoma migration and EMT through the transcriptional repression of *S100A14*, which is notably overexpressed in thyroid tumors and inversely correlated with *ZHX2* levels [24]. In colorectal cancer, according to one study, *S100A4* is substantially expressed and linked to distant metastases, brain invasion, lymph node involvement, and advanced TNM stage, whereas reduced *S100A14* expression facilitates EMT, proliferation, migration, and invasion [25]. Despite these findings, the molecular mechanisms underlying *S100A14*’s function in pancreatic cancer remain insufficiently characterized, highlighting the need for further investigation. In this study, we confirmed that *S100A14* is upregulated in PC cells. Functional assays demonstrated that knockdown of *S100A14* significantly inhibited proliferation, migration, invasion, and EMT of PC cells.

A previous study has identified a functional link between *S100A14* and *S100A16*, it is known that two S100 protein family members may form heterodimers [26,27]. *S100A16* has been extensively studied in PC, where it is aberrantly overexpressed and associated with poor patient prognosis. *S100A16* has also been extensively studied in PC, with accumulating evidence highlighting its oncogenic role. Li et al. reported that *S100A16* is aberrantly overexpressed in PDAC, correlating with poor patient prognosis. Mechanistically, *S100A16* promotes EMT by upregulating TWIST1 and activating the STAT3 signaling pathway, thereby facilitating tumor metastasis [28]. Similarly, Fang et al. demonstrated that *S100A16* significantly enhances PC cell proliferation, invasion, and migration through fibroblast growth factor 19 (FGF19)-dependent stimulation of the ERK1/2 and AKT pathways. Knockdown of *S100A16* induced cell cycle arrest and apoptosis, underscoring its potential as a therapeutic target [29]. Both *S100A14* and *S100A16* belong to the S100 protein family and can form heterodimers. Our findings reveal that *S100A14* and *S100A16* have a protein-protein interaction, and modulation of *S100A14* expression alters *S100A16* protein levels post-transcriptionally without affecting its mRNA expression, whereas overexpression of *S100A16* does not reciprocally influence *S100A14* levels. Functionally, *S100A16* overexpression promotes PC cell migration, invasion, and EMT, and inhibits cell apoptosis, effects that are reversed by *S100A14* knockdown, suggesting a hierarchical regulatory axis critical for PC progression.

Cellular homeostasis is crucially regulated by the tumor suppressor p53 via controlling the expression and stability of downstream targets such as p21 [30]. Its interaction with S100 family proteins further suggests a complex regulatory network influencing cancer invasion and migration, highlighting significant research potential. Previous studies have demonstrated that overexpression of *S100A14* enhances migration and invasion in esophageal squamous cell carcinoma, with its regulation of matrix metalloproteinase 2 (MMP2) being dependent on functional p53 [31]. Specifically, p53 represses MMP2 transcription, indicating that *S100A14* promotes tumor invasiveness through a p53-dependent mechanism. In PC, Chen et al. reported that ZWINT interacts with p53, facilitating its ubiquitination and degradation, thereby inhibiting the p53/p21 signaling axis and promoting cell proliferation and cell cycle progression [32]. The current investigation revealed that *S100A14* decreases p53 protein stability, suppressing both its expression and transcriptional activity, which attenuates p53 tumor suppressive functions. Furthermore, *S100A16* overexpression also inhibits p53 and its downstream effector p21, yet simultaneous knockdown of *S100A14* reverses this inhibition. These findings suggest that *S100A14* and *S100A16* have a protein-protein interaction and coordinately modulate the *p53/p21* axis, influencing the malignant phenotype of PC cells.

However, this study still has limitations: it only verifies the function of *S100A14* at the cellular level (e.g., proliferation, migration, and invasion assays), without establishing pancreatic cancer nude mouse models or orthotopic models, and lacks tumor-bearing mouse experiments to verify the role of the *S100A14/S100A16/p53* axis in tumor growth or metastasis *in vivo*; clinical samples only rely on data from public databases such as TCGA and GSE22780, with no inclusion of solid clinical samples from pancreatic cancer patients—it neither verifies the expression levels and tissue-level expression patterns of S100A14/S100A16 in cancer tissues and adjacent tissues via techniques like immunohistochemistry (IHC) nor analyzes their association with patients’ clinicopathological characteristics (e.g., tumor stage, differentiation degree), which significantly impairs the clinical translational value of the study conclusions, and the prognostic value of *S100A14* has not been verified through independent multi-center cohorts; at the molecular mechanism level, conclusions regarding *p53* protein stability remain vague, with no investigation into whether it involves *MDM2*-mediated ubiquitination or related mechanisms, and the specific molecular events (such as details of ubiquitination and phosphorylation modifications) underlying *S100A14*-mediated stabilization of *S100A16* and the interaction between *S100A16* and *p53* are also unclear. Future studies need to use *in vivo* xenograft/orthotopic tumor models to verify the impact of the *S100A14/S100A16/p53* axis on tumor growth and metastasis, validate the study conclusions and the prognostic value of *S100A14* through large-sample clinical cohorts and independent multi-center cohorts, conduct in-depth investigations into *MDM2*-mediated ubiquitination or related mechanisms in the regulation of *p53* protein stability, and further explore the molecular modification mechanisms of this regulatory axis, so as to lay a foundation for the development of therapeutic strategies targeting the *S100A14/S100A16/p53* axis in pancreatic cancer.

## 5 Conclusion

In summary, this study establishes S100A14 as a critical hub gene driving pancreatic cancer progression. Beyond its significant prognostic value and tumor-specific overexpression characteristics, *S100A14* functionally regulates the proliferation, migration, invasion, apoptosis, and EMT of pancreatic cancer cells. Mechanistically, *S100A14* interacts with *S100A16* protein and specifically stabilizes *S100A16* protein through post-translational regulatory mechanisms without affecting its mRNA expression. Crucially, this S100A14-S100A16 axis actively suppresses the p53 tumor suppressor pathway. S100A14 knockdown destabilizes S100A16 protein, subsequently restoring p53 protein stability, transcriptional activity, and expression of its downstream effector p21. These findings elucidate a novel axis involving S100A14, S100A16, and p53 that contributes to PC malignancy. From a translational perspective, this identified axis holds promising implications for developing targeted therapeutic strategies against pancreatic cancer—for instance, it could serve as a potential drug target to restore *p53*-mediated tumor suppression or as a prognostic biomarker to stratify high-risk patients. However, further studies are urgently needed to validate these findings: preclinical investigations using *in vivo* models (such as PC patient-derived xenografts or genetically engineered mouse models) should confirm the functional relevance of the *S100A14-S100A16-p53* axis in tumor progression, while clinical cohort studies are required to verify its prognostic and predictive value in larger patient populations.

## Data Availability

The datasets used and/or analyzed during the current study are available from the corresponding author upon reasonable request.
